# Equilibrium, Kinetic and Thermodynamic Study of Removal of Eosin Yellow from Aqueous Solution Using Teak Leaf Litter Powder

**DOI:** 10.1038/s41598-017-12424-1

**Published:** 2017-09-22

**Authors:** Emmanuel O. Oyelude, Johannes A. M. Awudza, Sylvester K. Twumasi

**Affiliations:** 1 Department of Chemistry, Kwame Nkrumah University of Science and Technology, Kumasi, Ghana; 2 Faculty of Public Health, Catholic University College, Fiapre, Sunyani, Ghana; 3 Department of Applied Chemistry and Biochemistry, University for Development Studies, Tamale, Ghana

## Abstract

Low-cost teak leaf litter powder (TLLP) was prepared as possible substitute for activated carbon. The feasibility of using the adsorbent to remove eosin yellow (EY) dye from aqueous solution was investigated through equilibrium adsorption, kinetic and thermodynamic studies. The removal of dye from aqueous solution was feasible but influenced by temperature, pH, adsorbent dosage and contact time. Variation in the initial concentration of dye did not influence the equilibrium contact time. Optimum adsorption of dye occurred at low adsorbent dosages, alkaline pH and high temperatures. Langmuir isotherm model best fit the equilibrium adsorption data and the maximum monolayer capacity of the adsorbent was 31.64 mg g^−1^ at 303 K. The adsorption process was best described by pseudo-second order kinetic model at 303 K. Boundary layer diffusion played a key role in the adsorption process. The mechanism of uptake of EY by TLLP was controlled by both liquid film diffusion and intraparticle diffusion. The values of mean adsorption free energy, E (7.91 kJ mol^−1^), and standard enthalpy, ΔH° (+13.34 kJ mol^−1^), suggest physical adsorption. The adsorption process was endothermic and spontaneous. Teak leaf litter powder is a promising low-cost adsorbent for treating wastewaters containing eosin yellow.

## Introduction

The colouration of dye-containing wastewater makes it aesthetically unacceptable^1^ though it may sometimes not be lethal to the environment. Other challenges wastewaters containing dyes may pose include: stability to irradiation and heat, slow biodegradation, obstruction of light penetration of water bodies and toxicity to aquatic organisms^2,3^. It is therefore important to treat wastewaters before disposal to avoid negative impact on the environment. The factors generally considered for using different techniques to treat dye-contaminated wastewaters include: safety, efficiency and budget^4^. The distinct merits and demerits of each of these techniques were concisely presented by Ashfaq & Khatoon^5^.

Eosin yellow (EY) is an anionic xanthen dye which is widely used for producing ink, pharmaceutical and textile products^6^. The dye causes eye and skin irritation^7^, inhibits protein-protein interaction^8^ and triggers geno-toxicity in man^9^. It is therefore vital that any wastewater containing EY be properly treated before disposal.

Adsorption, using activated carbon or graphene, is a popular physicochemical technique for treating dye-containing wastewaters because of its simplicity and efficacy of operation. The merits of adoption technology for wastewater treatment were expounded by Kanchi *et al*.^10^ and Yagub *et al*.^11^. However, commercial activated carbon and graphene are expensive, possess poor selectivity properties and require expensive regeneration after their adsorptive power is exhausted^12^. These challenges prompted researchers to investigate effective and cheaper adsorbents as substitutes for these materials.

There is on-going research into the development of smart systems for environmental applications^13,14^ including removal of dyes. The hindrance to their commercial development hinges on complex steps required and difficulty in scaling up^15^. Carbon nitride (CN) is used to produce 2D structures similar to graphene and with expansive applications^16,17^. The motivation is the enormous nitrogen-containing functional groups permitting assemblage of differs materials with exciting characteristics and applications. In practice, however, limited smart systems have been successfully assembled from nanostructured CN materials^12^.

Recently, Zhang and his co-workers^12^ hydrolyzed bulk CN in alkaline medium to prepare interfacial functionalized CN nanofibers. The materials were adjudged to possess high capacity, high selectivity, low energy-consuming regeneration, low toxicity, high versatility and moderate cost among other benefits. They were converted into 3D hydrogel network and applied to smart assembly systems with great success. Furthermore, they have been touted to pave way for better and cheaper treatment of dye-contaminated wastewaters, environmental remediation and selective extraction. The veracity of these claims cannot be independently established until the materials are available for use commercially.

Teak, *Tectona grandis*, is an important tree planted in the tropics on account of the attractiveness and durability of its wood. Its large broad leaves are shed mainly during the dry season generating enormous biomass. Leaf litter accounts for at least 70% of total teak litter fall^18^. Furthermore, Ojo *et al*. revealed that a monoculture teak plantation in Nigeria may produce up to 9,000 kg ha^−1^ litter per annum^19^. This vast quantity of biomass is currently unexploited and considered a nuisance.

A few non-conventional adsorbents studied have been shown to be promising for removing EY from aqueous solutions. Examples include: chitosan hydrobeads^20^, chitosan nanoparticles^21^, ethylenediamine modified chitosan^22^, de-oiled soya^23^ and bottom ash^6^. This study investigated the practicability of using low-cost adsorbent from teak leaf litter for removing EY in aqueous solution. The study includes: equilibrium adsorption, kinetics and thermodynamics studies.

## Results

### Characterization of TLLP

The moisture, volatile matter, ash and fixed carbon content of the adsorbent were: 3.88, 71.67, 7.51 and 16.94%, respectively. The values of matter soluble in water and matter soluble in acid were 4.77 and 6.97%, respectively. The adsorbent was slightly acidic (pH 6.81); although the pH of point of zero charge (pH_pzc_) was slightly basic with a value of 7.33. The bulk density, surface area and iodine number of TLLP were: 0.31 g cm^−3^, 20.87 m^2^ g^−1^ and 28.67 mg g^−1^, respectively.

### Equilibrium Adsorption

The variation in the initial concentration of EY did not exert any noticeable impact on the contact time required for the process to attain equilibrium (see Supplementary Fig. S1). The uptake of the dye from aqueous solution by TLLP was very rapid within the first 10 min and then slowed down considerably before gradually attaining equilibrium at 120 min. The variation in the dosage of TLLP significantly influence the quantity of EY removed from aqueous solution (see Supplementary Fig. S2). The dye removed from solution increased from 22.16% to 74.88% as the adsorbent dosage was raised from 1 to 12 g L^−1^. However, actual adsorption density decreased from 11.08 mg g^−1^ to 3.12 mg g^−1^ for the same variation in adsorbent dosage.

The removal of EY from aqueous solution by TLLP was significantly affected by pH of dye solution (see Supplementary Fig. S3). The uptake of dye was inversely proportional to the pH of the dye solution because the quantity of dye removed decreased from 9.96 mg g^−1^ at pH 3 to just 0.85 mg g^−1^ at pH 8. The removal of EY from aqueous solution by TLLP was favoured at high temperatures (see Supplementary Fig. S4). Adsorption capacity increased from 43.10 mg g^−1^ to 44.54 mg g^−1^ when temperature was raised from 298 K to 313 K.

### Adsorption isotherm

**Figure 1:**
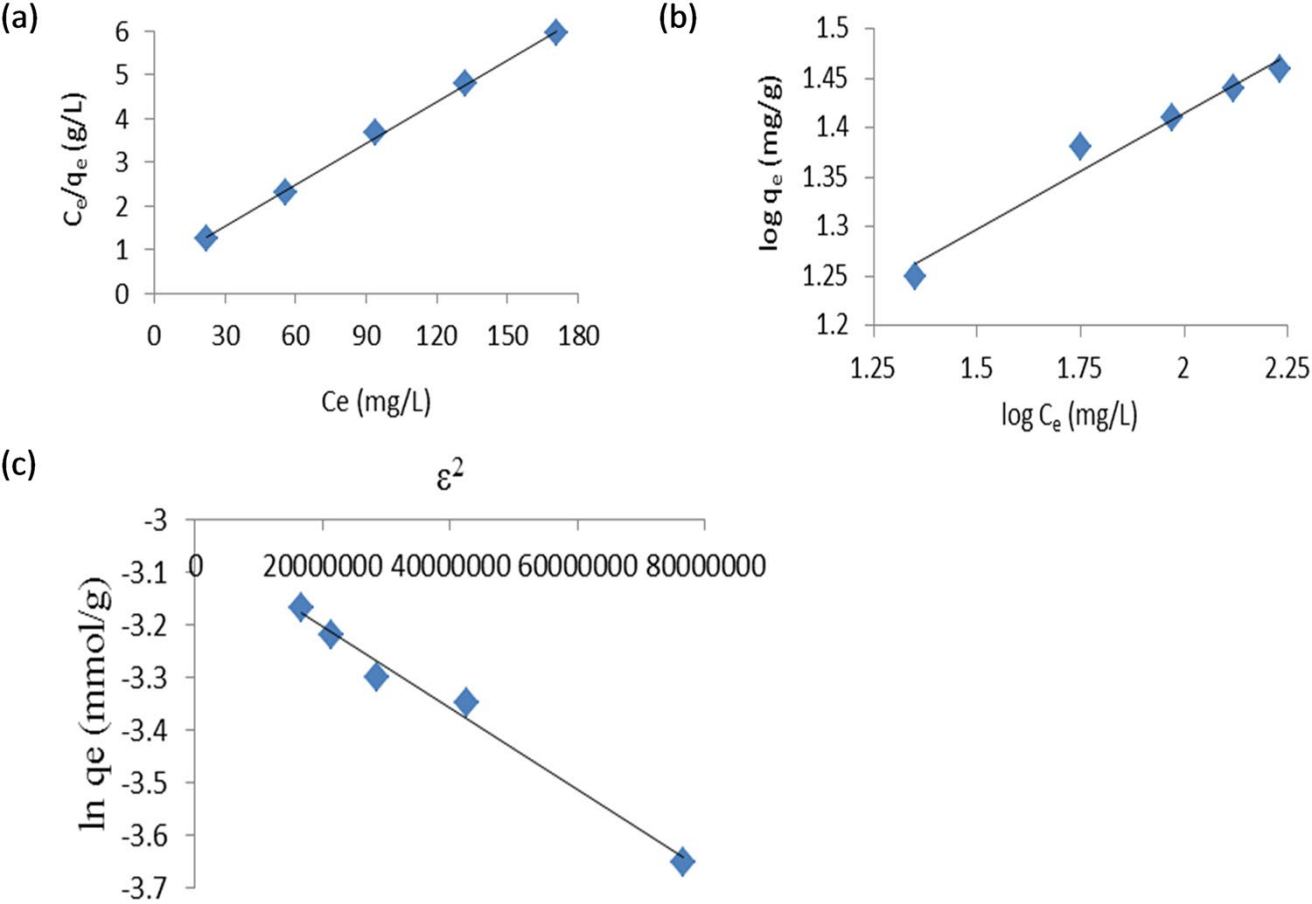
Linear plots of Langmuir (**a**), Freundlich (**b**) and Dubinin-Radushkevich (**c**) isotherms for removal of EY by TLLP.

The original data used for the linear plots of the adsorption isotherms are shown in Table 1. The linear plots of Langmuir, Freundlich and Dubinin-Radushkevich isotherms are presented in Fig. 1. The adsorption equilibrium data was best described by the Langmuir isotherm (R^2^ = 0.999). The equation generated from the linear for Langmuir isotherm plot in Fig. 1(a) was:

1 $$\frac{{C}_{e}}{{q}_{e}}=0.0316\frac{1}{{q}_{m}}+0.5872$$

from which the maximum monolayer adsorption capacity (q_m_) of TLLP was found to be 31.64 mg g^−1^. The value of separation factor (R_L_) was 0.085 implying favourable adsorption.

**Table 1:** Data for plotting adsorption isotherms.

C_o_ (mg L^−1^)	C_e_ (mg L^−1^)	q_e_ (mg g^−1^)	C_e_/q_e_ (g L^−1^)	log q_e_ (mg g^−1^)	log C_e_ (mg L^−1^)	ln C_e_ (mg L^−1^)
40	22.34	17.66	1.27	1.25	1.35	3.11
80	55.86	24.14	2.31	1.38	1.75	4.02
120	94.23	25.77	3.66	1.41	1.97	4.55
160	132.43	27.57	4.80	1.44	2.12	4.89
200	171.17	28.83	5.94	1.46	2.23	5.14

Note: Volume of EY = 100 mL, mass of TLLP = 0.1 g, pH of EY solution = 3 ± 0.10 and average room temperature = 303 ± 1 K.

The equation generated from the linear plot for Freundlich isotherm in Fig. 1(b) was:

2 $$\mathrm{log}\,{q}_{e}=0.2343\frac{1}{n}+0.9466$$

from which the values of Freundlich constant related to adsorbent capacity, K_F_, and the heterogeneity factor, 1/n, were determined as: 8.843 (mg g^−1^)(L mg^−1^)^1/n^, and 0.234, respectively. The equation produced from the linear plot for Dubinin-Radushkevich isotherm in Fig. 1(c) was:

3 $$\mathrm{ln}\,{q}_{e}=-(8.0\,\times \,{10}^{-9}){\varepsilon }^{2}-3.0509$$

from which the maximum capacity of TLLP was calculated as 32.74 mg g^−1^. The mean adsorption free energy, E, calculated from the Dubinin-Radushkevich isotherm plot, was 7.91 kJ mol^−1^ for this present study. The parameters, constants and correlation coefficients (R^2^) obtained from the three isotherm models are summarised in Table 2.

**Table 2:** Isotherm constants for the removal of EY from aqueous solution by TLLP.

**Langmuir Isotherm**	**K**_**L**_ **(L mg** ^**−1**^ **)**	**q**_**m**_ **(mg g** ^**−1**^ **)**	**R**_**L**_	**R** ^**2**^
	0.086	31.64	0.072	0.999
**Freundlich Isotherm**		**K**_**F**_ **(mg g** ^**−1**^ **)**	**1/n**	**R** ^**2**^
		8.843	0.234	0.970
**Dubinin-Radushkevich Isotherm**	**β (mmol** ^2^ **J** ^**−2**^ **)**	***q***_***DR***_ **(mg g** ^**−1**^ **)**	**E (kJ mol** ^**−1**^ **)**	**R** ^**2**^
	8.0 × 10^−9^	32.74	7.91	0.987

### Adsorption Kinetics

**Figure 2:**
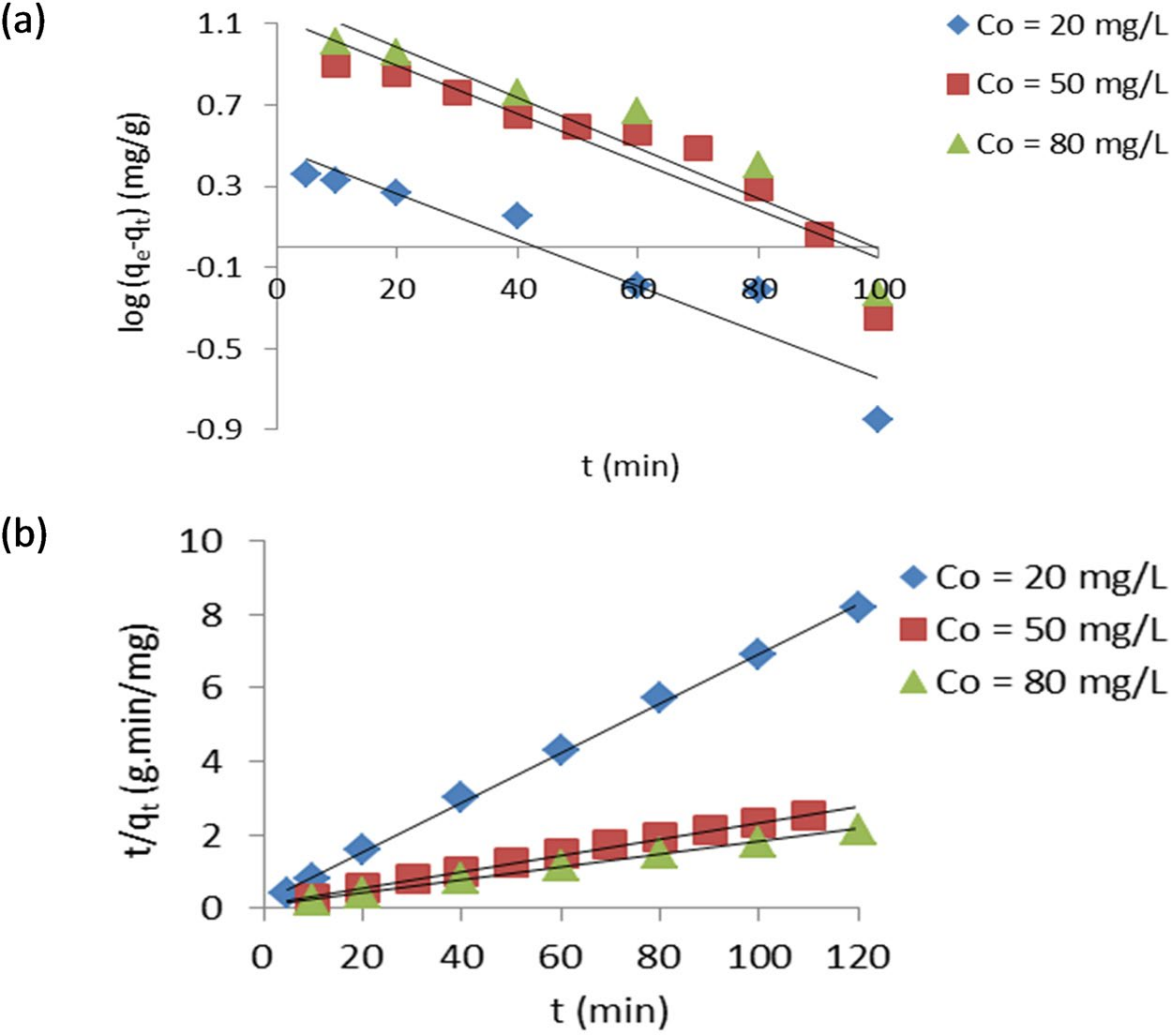
Pseudo-first order (**a**) and pseudo-second order (**b**) kinetic plots for removal of EY by TLLP.

**Figure 3:**
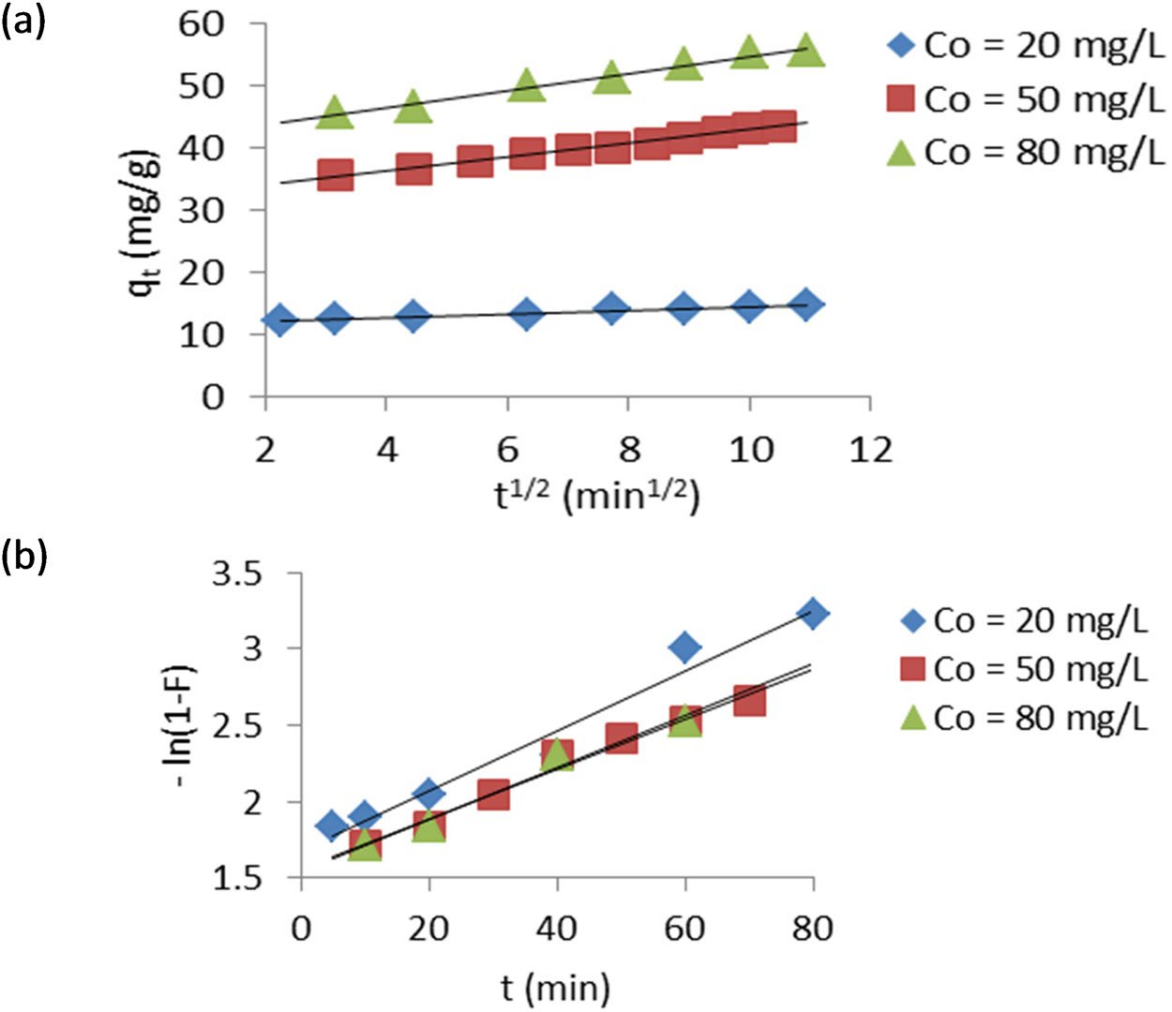
Intraparticle diffusion (**a**) and liquid film diffusion (**b**) plots for removal of EY by TLLP.

The original data used for the linear plots of the adsorption kinetics are presented as Supplementary Table S1. The linear plots of pseudo-first order and pseudo-second order at 303 K are presented in Fig. 2, while the intraparticle diffusion and liquid film diffusion models at 303 K are shown in Fig. 3. The pseudo-first order kinetic model with R^2^ values between 0.872 and 0.907 could not be used to describe the adsorption process. However, the adsorption of EY by TLLP best fit the pseudo-second order model because the mean value of R^2^ for every initial EY concentration tested was 0.998.

The intraparticle diffusion and liquid film diffusion plots were used to assess the mode of diffusion of EY into TLLP. The two sets of plots were linear with R^2^ values generally greater than 0.960 suggesting the applicability of both models to the kinetic data. However, the plots do not pass through the origin. The parameters, constants and correlation coefficients (R^2^) obtained from the four kinetic models are summarised in Table 3.

**Table 3:** Kinetic constants for removal of EY from aqueous solution by TLLP at 303 K.

Kinetic Models	Co (mg g^−1^)		
	20	50	80
**Pseudo-First Order**			
q_e_, mg g^−1^	3.102	13.64	17.25
k_1_, g/mg.min	0.026	0.027	0.029
R^2^	0.907	0.872	0.888
**Pseudo-Second Order**			
q_e_,_exp_ mg g^−1^	14.63	43.65	55.84
q_e_,_cal_ mg g^−1^	14.79	44.84	57.47
k_2_, g/mg.min	0.024	0.004	0.004
h, mg g^−1^.min	5.361	8.446	11.89
R^2^	0.999	0.998	0.998
**Intraparticle Diffusion**			
k_id_, mg g^−1^.min^1/2^	0.280	1.108	1.382
C, mg g^−1^	11.61	31.84	40.95
R^2^	0.983	0.988	0.991
**Liquid Film Diffusion**			
K_fd_, 1/min	0.020	0.016	0.017
C, mg g^−1^	1.681	1.553	1.535
R^2^	0.970	0.981	0.976

### Adsorption Thermodynamics

The values of thermodynamic parameters between 298 and 313 K are collated in Table 4. The values of standard free energy (ΔG°) ranged from −1.50 to −1.93 kJ mol^−1^ while standard enthalpy (ΔH°) and standard entropy (ΔS°) were 13.34 kJ mol^−1^ and 0.34 kJ mol^−1^ K^−1^, respectively. The values of ΔH° and ΔS° were obtained from supplementary Fig. S5.

**Table 4:** Thermodynamic parameters for the removal of EY from aqueous solution by TLLP.

ΔH° (kJ mol^−1^)	ΔS° (kJ mol^−1^ K^−1^)	ΔG° (kJ mol^−1^)			
		298 K	303 K	308 K	313 K
13.34	0.60	−1.50	−1.66	−1.80	−1.93

## Discussion

The surface area of TLLP was low which agrees with typical values for unmodified low-cost adsorbents with specific surface area that rarely exceed 50 m^2^ g^−1^ ^24,25^. Iodine number is used to assess the activity level of adsorbents. The very low value reported for the adsorbent corresponds with its low surface area. The bulk density was lower than approximately 0.50 g cm^−3^ reported for most commercial activated carbons^25,26^. Therefore, the surface of TLLP may need to be modified or the adsorbent converted to activated carbon to improve its characteristics and activity level.

The changes in the initial concentration of EY did not have any impact on the time required for the system to attain equilibrium. Equilibrium was attained at 120 min irrespective the initial concentration of the dye. On the basis of this result, subsequent experiments were agitated for 140 min to ensure that the adsorption process attained equilibrium.

The rapid removal of the dye at the initial stage is attributable to the presence of large number of free sites on the surface of TLLP that the dye molecules could attach to. The rate of uptake of dye was slow in the second stage because of significant reduction in the number of vacant sites on the surface of TLLP that dye molecules can bind to. There was equally a possibility of repulsion between dye molecules at the liquid-solid interface once all the sites on the adsorbent surface that dye molecules can bind to have been occupied. This observation is similar to the ones earlier reported by Chatterjee *et al*.^20^ and Mittal *et al*.^6^ who used chitosan hydrobeads and bottom ash, respectively to remove EY from aqueous solution.

The quantity of dye removed by the adsorbent generally increased as the adsorbent dose was increased. The observation is due to rapid superficial adsorption onto the surface of the adsorbent as TLLP to EY concentration ratio increased. However, actual adsorption capacity generally decreased with increase in adsorbent dosage due to aggregation of particles of the adsorbent. Similar observation has been reported by Huang *et al*.^22^.

The removal of EY from aqueous solution by TLLP was inversely proportional to the pH of the dye solution. The increased removal of anionic EY by TLLP at low pH values was due to electrostatic interaction between the positively charged binding sites on the surface of the adsorbent and the negatively charged dye ions. The magnitude of positive charge on the surface of the adsorbent reduced as pH increased and, hence, the reduction in the electrostatic interaction between the adsorbent and the dye ions. Similar results were reported by Mittal *et al*.^23^, Mittal *et al*.^6^ and Porkodi & Kumar^27^ who used de-oiled soya, chitosan hydrobeads and jute fiber carbon to remove EY from aqueous solutions.

The removal of EY from aqueous solution by TLLP was favoured at higher temperatures as adsorption capacity increased as temperature was raised. This suggests that the adsorption process was endothermic in nature. This observation conforms to the works of Mittal *et al*.^23^ and Mittal *et al*.^6^.

Adsorption isotherm models assist to estimate the capacity of an adsorbent as well as guides to design appropriate adsorption system^28^. The adsorption equilibrium data was best described by the Langmuir isotherm (R^2^ = 0.999). The calculated monolayer adsorption capacity of TLLP was 31.64 mg g^−1^ while the value of R_L_ was 0.085 suggesting that the removal of EY from aqueous solution by TLLP was favourable. The low adsorption capacity of the adsorbent is consistent with its low surface area and iodine number.

The adsorption capacity of TLLP is lesser than 399.04 mg g^−1^, 294.12 mg g^−1^ and 80.84 mg g^−1^ reported for tetraethylenepentamine modified sugarcane bagasse^29^, ethylenediamine modified chitosan^22^ and chitosan hydrobeads^20^, respectively. The very high adsorption capacity reported for tetraethylenepentamine modified sugarcane bagasse and ethylenediamine modified chitosan should be as a result of surface modification of the adsorbents. The capacity of TLLP is comparable to 36.18 mg g^−1^ and 31.49 mg g^−1^ reported for bottom ash^6^ and jute fibrecarbon^27^ but much higher than 9.82 mg g^−1^ and 3.33 mg g^−1^ reported for de-oiled soya^23^ and chitosan nanoparticles^21^, respectively.

The numerical value of 1/n (the heterogeneity factor) from the Freundlich isotherm plot was 0.234 implying that the adsorption process was favourable. The mean adsorption free energy, E, calculated from the Dubinin-Radushkevich isotherm plot, was 7.91 kJ mol^−1^ for this present study. This suggests that the adsorption process was physical in nature because its free energy was lesser than 8.00 kJ mol^−1^ ^30^.

The kinetics of removal of an adsorbate from aqueous solution assists one to understand the mechanism of the adsorption process and develop it for commercial application^31^. The kinetics of removal of EY from aqueous solution by TLLP is best described by the pseudo-second order model which is based on adsorption equilibrium capacity^32^. The closeness of experimentally determined and calculated values of q_e_ confirms the suitability of pseudo-second order kinetic as best fit for the adsorption kinetic.

The linear plots of the intraparticle and liquid film diffusion do not pass through the origin indicating that boundary layer diffusion was involved in the adsorption process^33^. Therefore, neither intraparticle diffusion nor liquid film diffusion was the sole rate-determining step. This indicates that both liquid film diffusion and intraparticle diffusion jointly control the removal of EY from aqueous solution by TLLP.

The values of ΔG° were negative at the range of temperature studied implying that the adsorption process was spontaneous and thermodynamically favourable. The positive value of ΔH° (+13.34 kJ mol^−1^) proved that the adsorption process was physical and endothermic in nature^34,35^. The positive value of ΔS° was a reflection of the increased randomness at the TLLP-EY solution interface due to the affinity of the adsorbent for the dye^36^.

## Conclusions

Low-cost TLLP was prepared from abundant but overlooked TLL. The low surface area and iodine number of the adsorbent culminates in its low activity level. The adsorbent may need to be modified or converted to activated carbon to improve its characteristics and activity level. The removal of EY from aqueous solution by TLLP was feasible but influenced by temperature, pH, adsorbent dosage and contact time. Variation in the initial concentration of dye exerted no influence on the equilibrium contact time of 120 min. Optimum adsorption of dye occurred at low adsorbent dosages, alkaline pH and high temperatures.

Langmuir isotherm model best fit the equilibrium adsorption data at 303 K. The calculated maximum capacity of TLLP as adsorbent for EY was 31.64 mg/g which is good for an unmodified low-cost adsorbent. The adsorption process was best described by pseudo-second order kinetic model at 303 K. Boundary layer diffusion played a key role in the adsorption process. The mechanism of uptake of EY by TLLP was jointly controlled by both liquid film diffusion and intraparticle diffusion but neither was the rate controlling step. The adsorption process was endothermic and spontaneous. The values of standard enthalpy, ΔH° (+13.34 kJ mol^−1^), and mean adsorption free energy, E (7.91 kJ mol^−1^) suggest physical adsorption. TLLP is a promising low-cost adsorbent for treating wastewaters contaminated by EY.

## Materials and Methods

### Materials

Teak leaf litter (TLL) was collected from the campus of University for Development Studies, Navrongo, Ghana. It was cleaned with copious quantity of tap water, rinsed with distilled water, air-dried for ten days and pulverized. The powdered TLL was washed with distilled water until the wash water was colorless, filtered and dried for 8 h in an oven (Selecta, Barcelona, Spain) set at 105 °C. The TLL was pulverised again and sieved to obtain average particle size below 210 µm. The adsorbent was transferred into a bottle, corked and labeled as teak leaf litter powder (TLLP).

TLLP was characterized by determining moisture content, volatile matter, ash and fixed carbon (by difference) using the method described by Jeyakumar & Chandrasekaran^37^. Moisture content was determined by drying TLLP in an oven at 105 ± 1 °C for 8 h. The moisture content was calculated as the percentage of the difference between the initial and final mass of the adsorbent. The volatile matter of the adsorbent was measured by drying in a muffle furnace the samples used for moisture content determination at 925 ± 2 °C for exactly 7 min. The percentage of the difference between the initial and final mass of the adsorbent was taken as the volatile matter content. The ash content was determined by transferring the samples used for volatile matter determination into a muffle furnace set at 1000 ± 2 °C for 4 h. The percentage of the difference between the initial and final mass of the adsorbent was taken as the ash content. The percentage fixed carbon of TLLP was calculated by subtracting the determined average values of percentage moisture content, volatile matter and ash content from 100.

Specific surface area was estimated using the method of Sears, Jr.^38^. Into a conical flask containing 1 g of adsorbent was added 50 mL of 0.1 M HCl. 20 g of NaCl was added and the volume of the solution was adjusted to 100 mL at pH between 3 and 3.5. 0.1 M NaOH solution was then titrated against the contents of the flask to raise its pH to 4. The volume (V, mL) of the 0.1 M NaOH required to raise the pH of the contents of the flask from pH 4 to pH was then recorded. The specific surface area (S, m^2^ g^−1^) of the adsorbent was then estimated from the relationship:

4 $$S=32V-25$$

The pH of the adsorbent was measured using pH meter initially calibrated at pH 4 and pH 7^39^. The pH of point of zero charge (pH_pzc_) using the method described by Hameed *et al*.^40^. Iodine number, bulk density, and matter soluble in water and acid were determined using standard procedures earlier described extensively by other researchers^41–43^.

All chemicals used were of analytical grade. The stock solution of dye prepared was 1,000 mg L^−1^ from which the working solutions were prepared as needed. A linear calibration plot of the dye (refer to Supplementary Fig. S6) was prepared and used to determine the concentration of residual dye after TLLP interacted with aqueous EY solution.

### Equilibrium adsorption

Equilibrium adsorption experiments were conducted at average room temperature of 303 ± 1 K. Predetermined volume of dye solution was added to 250 mL stoppered Erlenmeyer flasks containing appropriate mass of TLLP and agitated on mechanical shaker (HS 260, Ika-Werk GMBH, Germany) at 120 rpm. Portions of residual dye samples were removed from the flasks periodically and centrifuged (Selecta, Barcelona, Spain) at 2000 rpm for 5 min. The absorbance of the residual dye was read at 518 nm using uv/visible spectrophotometer (Baloworld Scientific Limited, England). The residual dye, q_t_ (mg g^−1^), q_e_ (mg g^−1^) or R (%), was calculated from the equations below.

5 $${q}_{t}=\frac{({C}_{o}-{C}_{t})V}{w}$$

6 $${q}_{e}=\frac{({C}_{o}-{C}_{e})V}{w}$$

and

7 $$R=\frac{({C}_{o}-{C}_{e})V}{w}$$

where, C_o_ (mg L^−1^) is the initial concentration of dye, C_t_ (mg L^−1^) is the concentration of dye at time, t (min), C_e_ (mg L^−1^) is the final concentration of dye, V (L) is the volume of dye solution and w (g) is the mass of TLLAC. The experiments were conducted in triplicates and the average value recorded. Any individual value that deviated from the average value by more than 5% was repeated.

The effects of contact time and initial concentration of EY solution were conducted using 1 g of adsorbent and 100 mL of dye solution. The initial concentrations of dye considered were: 20, 50 and 80 mg L^−1^. The impact of adsorbent dosage on adsorption was studied by fixing initial concentration and volume of EY at 20 mg L^−1^ and 100 mL, respectively. The mass of TLLP was varied from 0.1 g to 1 g. The impact of pH of dye solution on adsorption was studied by fixing the mass of adsorbent, volume and initial concentration of dye at 0.2 g, 50 mL and 20 mg L^−1^, respectively. The effect of temperature was studied at 5 K intervals from 303 to 318 K. The mass of adsorbent, initial concentration, volume and pH of EY were fixed at 0.1 g, 40 mg L^−1^, 100 mL and 3, respectively.

### Adsorption Isotherm

Data used for describing the isotherm was obtained by fixing the mass of adsorbent, temperature, volume and pH of EY at 0.1 g, 303 K, 100 mL and pH 3, respectively. However, the initial concentration of the dye solution was varied from 40 to 200 mg L^−1^.The equilibrium adsorption data was evaluated using Langmuir, Freundlich and Dubinin-Radushkevich isotherms. The linearised Langmuir equation^44^ is

8 $$\frac{{C}_{e}}{{q}_{e}}=\frac{1}{{q}_{m}}{C}_{e}+\frac{1}{{q}_{m}}\frac{1}{{K}_{L}}$$

where C_e_ (mg L^−1^) and q_e_ (mg g^−1^), respectively, is the concentration and mass of dye removed at equilibrium per unit mass of adsorbent, q_m_ (mg g^−1^) is the monolayer adsorption capacity of the adsorbent, and K_L_ (L mg^−1^) is the Langmuir constant related to the rate of adsorption. The values of q_m_ and K_L_ were determined from the plot of C_e_/q_e_ against C_e_.

The separation factor, R_L_, was used to explain the applicability of Langmuir isotherm to the adsorption data. R_L_ is given by:

9 $${R}_{L}=\frac{1}{1+{K}_{L}{C}_{o}}$$

where K_L_ (L mg^−1^) and C_o_ (mg L^−1^) are the Langmuir adsorption constant and the highest initial concentration of adsorbate, respectively.

Freundlich isotherm assumes multilayer adsorption with heterogeneous surface^45^. The linearised Freundlich adsorption equation is

10 $$\mathrm{log}\,{q}_{e}=\frac{1}{n}\,\mathrm{log}\,{C}_{e}+\,\mathrm{log}\,{K}_{F}$$

where q_e_ (mg g^−1^) is the mass of adsorbate removed at equilibrium per unit mass of adsorbent and C_e_ (mg L^−1^) is the concentration of adsorbate removed by adsorbent at equilibrium and K_F_ (mg g^−1^)(L mg^−1^)^1/n^ and 1/n are constants representing the adsorbent capacity and the heterogeneity factor, respectively. The values of 1/n and K_F_ were determined from the plot of log q_e_ against log C_e_.

Dubinin-Radushkevich isotherm^46^ was used to determine the mean free energy and nature of adsorption. The linear form of Dubinin-Radushkevich equation is

11 $$\mathrm{ln}\,{q}_{e}=\,\mathrm{ln}\,{q}_{DR}-\beta {\varepsilon }^{2}$$

where ${q}_{{DR}}$ (mg g^−1^) is the Dubinin-Radushkevich maximum monolayer adsorption capacity, β (mol^2^ J^−2^) is activity coefficient related to mean adsorption energy, and ε is the Polanyi potential which is calculated using the following relationship

12 $$\varepsilon =RT\,\mathrm{ln}(1+\frac{1}{{C}_{e}})$$

where R (8.314 J mol^−1^ K^−1^) is the gas constant, T (K) is temperature and C_e_ (mg L^−1^) is the concentration of adsorbate at equilibrium. The values of q_DR_ and β were determined from the plot of ln q_e_ against ε^2^. The mean free energy of adsorption, E (J mol^−1^), was then determined from the following equation

13 $$E=\frac{1}{\sqrt{2\beta }}$$

### Adsorption Kinetics

The kinetics experiments were conducted by fixing the initial concentration of EY at 20, 50 and 80 mg L^−1^. However, mass of TLLP, volume, temperature and pH of dye solution were fixed at 0.1 g, 100 mL, 313 K and pH 3, respectively. The rate of uptake of dye by the adsorbent was monitored at predetermined uniform time intervals until equilibrium was attained.

The adsorption kinetic data was assessed using four models at 303 K. The pseudo-first order equation^47^ is:

14 $$\mathrm{log}({q}_{e}-{q}_{t})=\,\mathrm{log}\,{q}_{e}-{k}_{1}t$$

where, q_e_ (mg g^−1^) and q_t_ (mg g^−1^) are the quantity of EY removed at equilibrium and time, t (min), respectively; and k_1_ (min^−1^) is the pseudo-first order rate constant. The values of k_1_ and q_e_ were determined from the plot of log (q_e_ − q_t_) against t. This model tends to be valid during the initial period of adsorption when the rate of removal of adsorbate by adsorbent is fast.

The pseudo-second order equation^32^ tends to be applicable over the entire duration of an adsorption process. The equation is

15 $$\frac{t}{{q}_{t}}=\frac{1}{{k}_{2}{q}_{e}^{2}}+\frac{1}{{q}_{e}}t$$

where, q_t_ (mg g^−1^), q_e_ (mg g^−1^) and t (min) are as defined earlier and k_2_ (g mg^−1^ min^−1^) is the pseudo-second order rate constant. The values of q_e_ and k_2_ were determined from the plot of t/q_t_ against t. The initial rate of adsorption, h, was calculated from the following relationship:

16 $$h={k}_{2}{q}_{e}^{2}$$

The value of k_2_ is normally inversely proportional to the initial concentration of adsorbate.

The adsorbate ions or molecules adsorbed on the surface of adsorbent particles may move from the surface into the pores. The influence of pore diffusion was studied by applying intraparticle diffusion equation^48^

17 $${q}_{t}={k}_{id}{t}^{1/2}+C$$

where, q_t_ (mg g^−1^) and t (min) are as earlier defined, k_id_ (mg g^−1^ min^−1/2^) is the intraparticle diffusion rate constant and C is a constant pertaining to boundary layer. The values of k_id_ and C were obtained from the plot of q_t_ against t^1/2^.

Liquid film diffusion^49^ was applied to aid in the clarification of the rate controlling stage of the adsorption mechanism. The equation is

18 $$\mathrm{ln}\,(-F)=-{k}_{fd}t+C$$

where, F = q_t_/q_e_, k_fd_ (min^−1^) is the liquid film diffusion rate constant, t (min) is time, and C is a constant related to the boundary layer. The value of k_fd_ was determined from the plot of −ln(1 − F) against t.

### Adsorption Thermodynamics

The experiments on thermodynamics of adsorption were similar to those on the effects of temperature described earlier. However, the flask containing the adsorbent-dye mixture was agitated in a water bath (Heizbad HB, Heidolph, Germany) earlier set at a predetermined temperature. The rate of removal of EY by TLLP was monitored until equilibrium was attained.

Standard enthalpy (ΔH°, kJ mol^−1^), standard entropy (ΔS°, kJ mol^−1^ K^−1^), and standard free energy (ΔG°, kJ mol^−1^) were determined by conducting experiments at various temperatures. The following relationships were employed:

19 $${\rm{\Delta }}{G}^{\circ }={\rm{\Delta }}{H}^{\circ }-T{\rm{\Delta }}{S}^{\circ }$$

20 $${\rm{\Delta }}{G}^{\circ }=-RT\,\mathrm{ln}\,{K}_{d}$$

21 $${K}_{d}=\frac{{q}_{e}}{{C}_{e}}$$

22 $$\mathrm{ln}\,{K}_{d}=\frac{{\rm{\Delta }}{S}^{\circ }}{R}-\frac{{\rm{\Delta }}{H}^{\circ }}{RT}$$

where, R is the gas constant (8.314 J mol^−1^ K^−1^), T (K) is temperature, K_d_ is the distribution coefficient, q_e_ (mg g^−1^) is the quantity of adsorbate removed at equilibrium and C_e_ (mg L^−1^) is the quantity of adsorbate remaining in solution at equilibrium. Equation (16) was used to determine the values of ΔG° at various temperatures. The values of ΔH° and ΔS° were estimated from the plot of ln K_d_ versus T^−1^ (see Supplementary Fig. S5).

## Electronic supplementary material

Supplementary Information
