# Supplementary material for: Equilibrium, Kinetic and Thermodynamic Study of Removal of Eosin Yellow from Aqueous Solution Using Teak Leaf Litter Powder
Source: Sci Rep. 2017 Sep 22;7:12198. doi: 10.1038/s41598-017-12424-1 (PMC5610235; doi:10.1038/s41598-017-12424-1)
Supplement: Supplementary file 1 — Supplementary Information [file 41598_2017_12424_MOESM1_ESM.doc]

**Equilibrium, Kinetic and Thermodynamic Study of Removal of Eosin Yellow from Aqueous Solution Using Teak Leaf Litter Powder**

**Emmanuel O. Oyelude^1, 3*^, Johannes A.M. Awudza^1^ and Sylvester K. Twumasi^2^**

^1^Department of Chemistry, Kwame Nkrumah University of Science and Technology, Kumasi, Ghana

^2^Faculty of Public Health, Catholic University College, Fiapre, Sunyani, Ghana

^3^Department of Applied Chemistry and Biochemistry, University for Development Studies, Tamale, Ghana

^*^emmanola@gmail.com


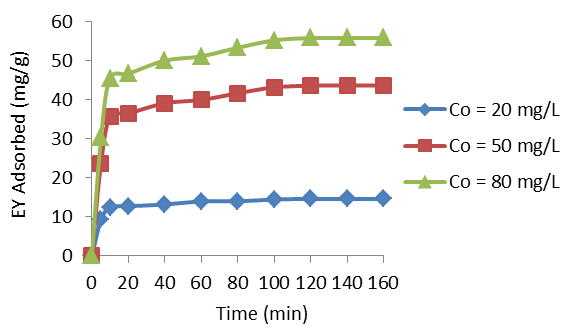


Figure S1 Effect of initial concentration of dye and contact time on removal of EY by TLLP


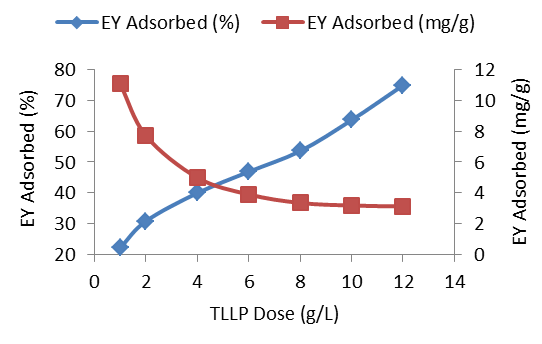


Figure S2 Effect of TLLP dose on removal of EY by TLLP


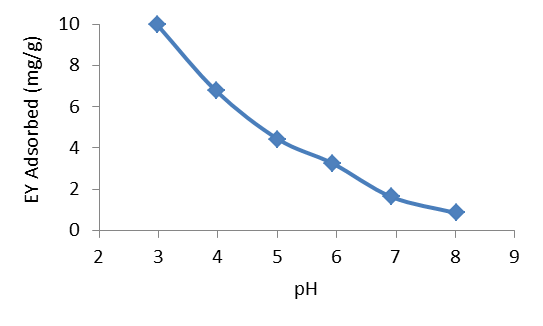


Figure S3 Effect of pH of dye solution on removal of EY by TLLP


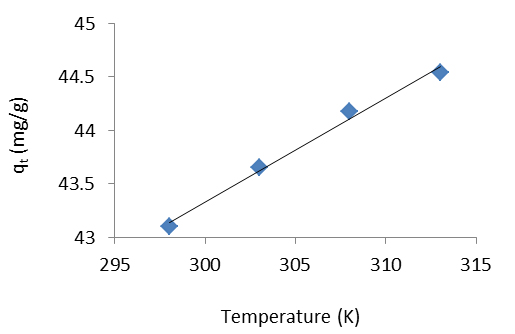


Figure S4 Effect of temperature of dye solution on removal of EY by TLLP


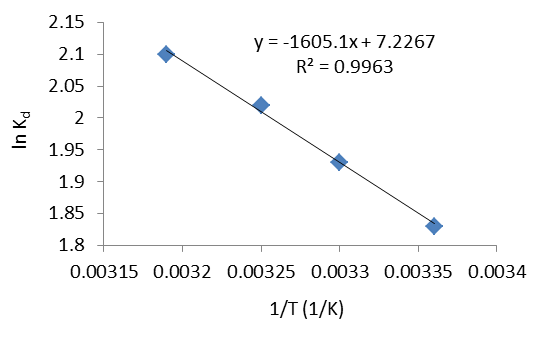


Figure S5 Plot of ln K_d_ versus 1/T for determination of ΔH° and ΔS°


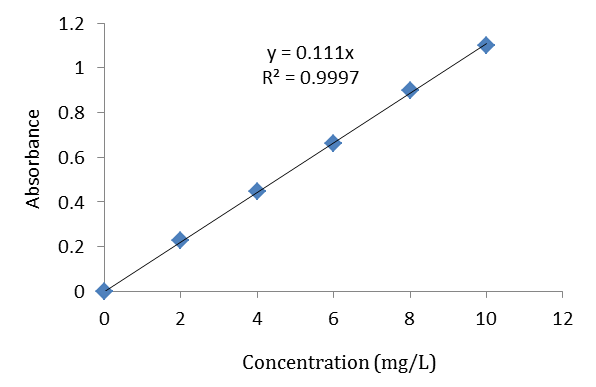


Figure S6 Calibration plot for dilute EY solution at 518 nm

Table S1 Data for plotting adsorption kinetics

| **t (min)** | **Ce** | **qt** | **qe - qt** | **log (qe – qt)** | **t/qt** | **t^1/2^** | **F = qt/qe** | **ln (1 – F)** | **Βt** | **- ln (1 – F)** |
| --- | --- | --- | --- | --- | --- | --- | --- | --- | --- | --- |
| C_o_ = 20 mg L^-1^ | |  |  |  |  |  |  |  |  |  |
| 5  10  20  40  60  80  100  120 | 7.68  7.50  7.24  6.77  6.02  5.98  5.51  5.37 | 12.32  12.50  12.76  13.23  13.98  14.02  14.49  14.63 | 2.31  2.13  1.87  1.40  0.65  0.61  0.14  0 | 0.36  0.33  0.27  0.15  -0.19  -0.21  -0.85 | 0.41  0.80  1.57  3.02  4.29  5.71  6.9  8.2 | 2.24  3.16  4.47  6.32  7.75  8.94  10.00  10.95 | 0.84  0.85  0.87  0.90  0.95  0.96  0.99 | -1.83  -1.90  -2.04  -2.30  -3.00  -3.22  -4.60 | 1.33  1.40  1.54  1.80  2.50  2.72  4.10 | 1.83  1.90  2.04  2.30  3.00  3.22 |
| C_o_ = 50 mg L^-1^ | |  |  |  |  |  |  |  |  |  |
| 10  20  30  40  50  60  70  80  90  100  110 | 14.37  13.47  12.07  10.86  10.23  10.00  9.41  8.29  7.51  6.8  6.35 | 35.63  36.53  37.93  39.14  39.77  40.00  40.59  41.71  42.49  43.20  43.65 | 8.02  7.12  5.72  4.51  3.88  3.65  3.06  1.94  1.16  0.45  0 | 0.90  0.85  0.76  0.65  0.59  0.56  0.49  0.29  0.06  -0.35 | 0.28  0.55  0.79  1.02  1.26  1.50  1.72  1.92  2.12  2.31  2.52 | 3.16  4.47  5.48  6.32  6.71  7.75  8.37  8.94  9.49  10.00  10.49 | 0.82  0.84  0.87  0.90  0.91  0.92  0.93  0.96  0.97  0.99 | -1.71  -1.83  -2.04  -2.30  -2.41  -2.52  -2.66  -3.22  -3.51  -4.60 | 1.21  1.33  1.54  1.80  1.91  2.02  2.16  2.72  3.01  4.10 | 1.71  1.83  2.04  2.30  2.41  2.52  2.66  3.22  3.51  4.60 |

Note:

Volume of EY: 100 mL

Mass of TLLP: 0.1000 g

pH: 3 ± 0.10

Average room temperature: 303 ± 1 K

Table S1 Data for plotting adsorption kinetics cont’d

| **t (min)** | **Ce** | **qt** | **qe - qt** | **log (qe – qt)** | **t/qt** | **t^1/2^** | **F = qt/qe** | **ln (1 – F)** | **Βt** | **- ln (1 – F)** |
| --- | --- | --- | --- | --- | --- | --- | --- | --- | --- | --- |
| C_o_ = 80 mg L^-1^ | |  |  |  |  |  |  |  |  |  |
| 10  20  40  60  80  100  120  140 | 34.43  33.28  29.94  28.84  26.65  24.75  24.16  24.16 | 45.47  46.72  50.06  51.16  53.35  55.25  55.84  55.84 | 10.27  9.12  5.78  4.68  2.49  0.59  0 | 1.01  0.96  0.76  0.67  0.40  -0.23 | 0.22  0.43  0.80  1.17  1.50  1.81  2.15 | 3.16  4.47  6.32  7.75  8.94  10.00  10.95 | 0.82  0.84  0.90  0.92  0.96  0.99 | -1.71  -1.83  -2.30  -2.52  -3.22  -4.60 | 1.21  1.33  1.80  2.02  2.72  4.10 | 1.71  1.83  2.30  2.52  3.22  4.60 |

Note:

Volume of EY: 100 mL

Mass of TLLP: 0.1000 g

pH: 3 ± 0.10

Average room temperature: 303 ± 1 K
